# Scrotal Lymphangioma – A Rare Cause of Scrotal Swelling

**Published:** 2013-01-01

**Authors:** Pradeep Kajal, Kamal Nain Rattan, Vivek S Malik, Vipin Garsa

**Affiliations:** Department of Pediatric Surgery, PGIMS Rohtak, Haryana.; Department of Pediatric Surgery, PGIMS Rohtak, Haryana.; Department of Anatomy, PGIMS Rohtak, Haryana.; Department of Anatomy, PGIMS Rohtak, Haryana.

**Keywords:** Lymphangioma, Scrotum, Child

## Abstract

Lymphangioma is an extremely rare cause of scrotal swelling. We are reporting such a tumor in a one and half year old child presenting with a painless, progressive scrotal swelling. The mass was evaluated and excised completely. Histopathology confirmed it as Lymphangioma.

## INTRODUCTION

Lymphangioma are congenital lymphatic hamartomas, 95% of which occur in neck and axilla [1]. The scrotum and retroperitoneum are unusual sites [2]. The scrotum is one of the rarest sites for lymphangioma. It can involve scrotal wall, tunics, testis, epididymis, spermatic cord or Colle’s fascia [3]. This is a report of scrotal lymphangioma with special emphasis on clinical, ultrasonographic, and histopathological findings. 

## CASE REPORT

An 18-month-old male child presented with painless, progressive enlargement of left hemiscrotum for last one year. Examination revealed a non-tender and soft mass in left hemi scrotum which was compressible with positive transillumination test. The left testis could be felt separately from the mass (Fig. 1). Patient underwent surgery after routine investigations. At operation a multilocular cystic mass measuring 5cmx5cm adherent to the tunics found. The left testis and spermatic cord were not involved (Fig. 2). Complete excision of the mass along with overlying skin was done. Suction drain kept at surgery was removed on 2nd postoperative day. Child was discharged on 3rd postoperative in good health. On histopathological examination, it was reported as cystic lymphangioma. No recurrence was noted at one year follow up. 

**Figure:**
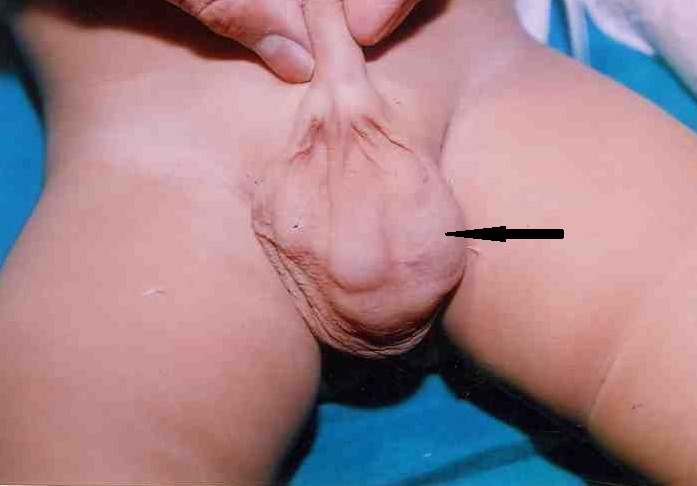
Figure 1: Clinical photograph showing left scrotal swelling (arrow) separate from left testis.

**Figure:**
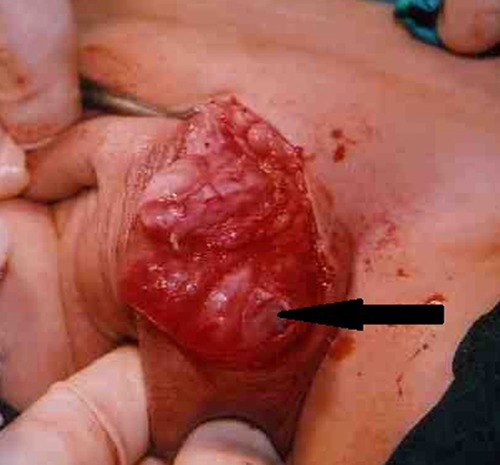
Figure 2: Cystic lymphangioma (arrow) arising from scrotal wall and adherent to the tunics.

## DISCUSSION

Lymphangioma results from inadequate drainage of lymph from sequestrated lymphatic vessels and are considered to be lymphatic hamartomas. Most of these are congenital due to paucity or atresia of the efferent lymphatics or lack of communication between lymphatics and venous channels [1]. These lesions can also be acquired because of obstruction of lymphatics after inflammation, trauma or degeneration [3]. 

Histologically, three types – capillary, cavernous and cystic are described. The cystic form is the most common variety. Half of these are recognized at birth, 90% are evident by the age of 2 years and 95% occur in the neck or axilla. Unusual sites are scrotum, retroperitoneum, intraperitoneum, gluteal region, mediastinum, groin, pelvis, mesentery, omentum and spleen [1, 4, 5]. Scrotum is a rare site for lymphangioma. Singh et al reported thirty two cases of cystic lymphangiomas in children, and only one was located in the scrotum [1]. Loberant et al estimated that less than fifty cases of scrotal cystic lymphangioma have been reported in literature till 2002 [6]. When scrotum is involved, it is usually misdiagnosed as hernia, hydrocele, varicocele, large epididymal cyst or acute scrotal conditions which may lead to inadequate treatment with a risk of recurrence [2, 5, 7]. Hurwitz et al reported seven cases of scrotal cystic lymphangioma over a period of ten years, all of which were misdiagnosed preoperatively [7]. Ultrasound scan usually shows a multicystic extra testicular mass with internal septae; internal echoes in the cysts due to debris and/or hemorrhage may be noted as a result of complication [6, 8]. The treatment consists of surgical excision of the entire mass along with the overlying skin. Incomplete excision can result in recurrence [7]. Scrotal lymphangioma though rare must be kept in mind while exploring any scrotal swelling.

## Footnotes

**Source of Support:** Nil

**Conflict of Interest:** None declared
